# A process evaluation of everyday life rehabilitation: a critical discourse analysis of housing staff perspectives

**DOI:** 10.3389/frhs.2026.1771613

**Published:** 2026-09-24

**Authors:** André Sjöberg, Linda Richter Sundberg, Maria Lindström

**Affiliations:** 1 Department of Epidemiology and Global Health, Umeå University, Umeå, Sweden; 2 Department of Psychology, Umeå University, Umeå, Sweden

**Keywords:** attitude of health personnel, community health services, group homes, leisure activities, personal recovery, qualitative research, rehabilitation, supported accommodation

## Abstract

**Background:**

Residents in supported accommodation often lead lonely and sedentary lives, with limited influence on everyday decisions. In addition, long-term outreach-based rehabilitative efforts and integrated care remain uncommon in this setting. The intervention, Everyday Life Rehabilitation (ELR), was developed specifically for this setting and has demonstrated robust effectiveness in an experimental trial. Yet implementation of similar interventions often meets resistance. This study examines how organisational discourse and contextual determinants shape the implementation of recovery-oriented interventions in supported accommodation.

**Method:**

This is an embedded process evaluation following a randomised controlled trial of ELR in Sweden. Focus group interviews were conducted with housing staff (*n* = 23) across seven supported accommodation units. Transcribed interviews were analysed using critical discourse analysis to examine how language, power relations, and organisational context shaped staff’s understandings of the implementation process.

**Findings:**

Our interpretation is that the core discourses among housing staff revealed how they diminished the intervention's perceived novelty and legitimised existing practices to cope with change. The discourses often constrained resident's potential to maintain control over collective resources in accordance with organisational norms. Across the corpus, staff discourses protected the boundary between insiders and outsiders, potentially generating tensions regarding collaboration, ownership, and power. Finally, vague leadership often blurred the lines of accountability, requiring staff and occupational therapists to negotiate responsibilities within organisational constraints.

**Conclusion:**

Discourse analysis can be a valuable methodology for understanding how implementation barriers emerge and are sustained in supported accommodation. We argue that the identified barriers did not reflect staff resistance to the intervention but rather how the implementation process was framed within the organisation. Our interpretation is that structural limitations, including vague leadership and unclear accountability for implementation, constrained staff agency and contributed to discursive strategies that legitimised resistance and gatekeeping.

## 1 Introduction

Three decades ago, Sweden implemented a major psychiatric reform, shifting the responsibility for supported accommodation and day care services for individuals with serious mental illness (SMI) from regional to municipal authorities (1). Although community-based mental health services have become common practice in many countries, research suggests they tend to reproduce the flaws of traditional psychiatric institutions (2, 3). Many residents remain socially isolated and struggle to engage in meaningful activities, resulting in lonely and sedentary lives (4, 5). Most of their activities are passive, lack purpose, and are confined to the residential facility, reflecting a form of occupational injustice (6). Dependence on staff further diminishes residents’ sense of autonomy, with many reporting that they feel constrained in making everyday decisions (7, 8). From an organisational perspective, staff working in supported accommodation have low levels of education and limited relevant training (1, 9), while managerial support is limited due to the prioritisation of administrative responsibilities (10, 11). In addition, fragmented collaboration between health and social services makes the delivery of integrated care difficult (12, 13). These organisational barriers foster an environment in which staff often lack the necessary conditions to effectively enable residents to engage in meaningful activities (14).

To address these challenges, Everyday Life Rehabilitation (ELR) was developed (15–18). It is a person-centred recovery- and activity-based approach that promotes personal recovery through engagement in meaningful everyday activities. Recent findings from a randomised controlled trial (RCT) demonstrate that ELR is effective in improving both personal recovery and quality of life (19). According to the updated Medical Research Council framework, process evaluations are essential in the development and evaluation of complex interventions (20). Their value is particularly evident in pragmatic trials, where it often is challenging to distinguish the intervention from contextual factors (21). Previous studies on recovery training programs for mental health staff have shown inconsistent results, largely due to implementational challenges (22). Key barriers include unequal power dynamics, limited resources, high staff turnover, and competing priorities (23–25). Yet explaining how these barriers influence outcomes requires mechanism-based analyses that account for the complexity of social context and human agency (26, 27).

The Swedish welfare system is highly decentralised, with regions and municipalities being responsible for different segments of the health system (28). This fragmentation makes service coordination difficult, contributes to health inequalities, and creates challenges for collaboration between professional groups (29, 30). Although Swedish municipalities are legally obliged to provide residents in supported accommodation with access to basic healthcare services and opportunities to participate in their local communities (31–33), access to integrated community-based rehabilitation services remains limited (34). Swedish-supported accommodation typically provides high levels of care 24/7 with limited emphasis on transition to ordinary housing, which corresponds to a type-1 facility in the STAX-SA taxonomy (35). It is also common in type-4 facilities, in the form of “satellite apartments,” where residents live more independently but remain in close proximity to staff (36). In many municipalities, there is a general shift away from the large-scale group homes established during the first wave of deinstitutionalisation to more outreach-based housing models (1).

Interventions function as strategic disruptions to the mechanisms that perpetuate problems within a system (37), while organisations operate as hegemonic structures that reproduce power relations to maintain operational stability. Accordingly, interventions such as ELR are not implemented in a vacuum; they challenge established routines, ideologies, and power relations embedded in practice and organisational structures. During implementation, established power relations are challenged, forcing actors to develop competing strategies for negotiating new organisational practices. These strategies are often discursive, employing narratives and discourses that reframe past events, present circumstances, and imaginaries of what could happen (38). In this study, the term actor refers to social entities, primarily individuals and groups, that are represented as having varying degrees of agency and influence. Given that recovery-oriented practices often require staff to redefine their roles as practitioners (39), successful implementation depends on how they operationalise these strategies.

Critical discourse analysis (CDA) offers a suitable framework for understanding these strategies (38, 40, 41). Its philosophical grounds enable not only a critical analysis of staff discourses but also a critique of the social conditions that shape them, raising awareness of how discourse reproduces ideology and how language functions as an active force in shaping social practices, identities, and power dynamics. The aim of this process evaluation is to explore housing staff's experiences of, and reasoning around, the implementation of ELR, with particular attention to how discourses, social dynamics, and contextual conditions influence both individual and collective behaviour. Adopting a critical perspective allows us to question taken-for-granted assumptions and make the interests and needs of different actors visible (41). By analysing staff discourses, this study seeks to uncover how meanings of roles, responsibilities, and the novel intervention are constructed and contested during the implementation process.

## 2 Materials and methods

This study is an embedded process evaluation within a larger cluster RCT evaluating the effectiveness of ELR (19). The full-scale RCT was ongoing between 2021 and 2024, with units stratified into four waves, each unit receiving ELR for 6 months. Data for the current study were collected through focus group interviews with housing staff following their unit's participation in the trial. Although qualitative interviews were not outlined in the original trial protocol (NCT05056415; ClinicalTrials.gov), they were conducted to better understand the implementation processes and contextual factors. Reporting was guided by the Consolidated Criteria for Reporting Qualitative Research (42). The analysis was conducted using Fairclough's dialectical–relational approach (40), which emphasises the relationship between language use and power dynamics (43, 44). This approach enables us to examine how explicit and implicit assumptions, resistance, and contextual constraints influenced the implementation process, and how implementation determinants emerge and are reproduced through discourse.

Recruitment for the focus groups was conducted using a purposive sampling strategy to achieve maximum variation in implementational outcomes (45). During this process, municipal contact persons were asked to nominate units that exemplified either smooth or problematic implementation processes. This selection strategy was intended to capture the full spectrum of experiences, discourses, and patterns related to the intervention (46). Recruitment occurred continuously throughout the trial period as units completed their participation in the RCT. As a result, the time elapsed between intervention completion and interview varies across units, with most interviews conducted approximately 6–12 months after participation ended.

Invitations were extended to a total of 11 contact persons via email and/or telephone, who were selected based on convenience and the availability of contact information. The categorisations of units as smooth or problematic were primarily for sampling and were not intended as analytical categories. Two of the analysts (ML and LRS) did not receive any information at all about which category each unit belonged to. Accordingly, the corpus was analysed at the unit level rather than stratified according to perceived implementation success.

### 2.1 Intervention

The implementational object in this study is the intervention, ELR, which has been described in previous publications (15, 16, 19). The target population of the intervention is individuals with SMI, with the target users being the housing staff, occupational therapists, and housing managers who deliver the intervention in the context of supported accommodation. The occupational therapist and resident collaboratively develop an individual plan, which staff are then expected to follow in their usual practice. Activities are individually chosen and tailored to each resident, varying in type, frequency, and intensity based on personal preferences and goals. The core function of the intervention is to enable residents to engage in self-selected everyday activities that they themselves perceive as meaningful, based on the hypothesis that such engagement improves both personal recovery and quality of life (19).

The intervention encourages residents to explore and gradually develop meaningful activities through real-life activity training, supported by key components of person-centred care in the CHIME framework (47). The intervention has been developed in different phases, in close collaboration with stakeholders (15). Although the intervention was not originally developed using the ERIC (Expert Recommendations for Implementing Change) framework, its components can be retrospectively mapped to ERIC's discrete implementation strategies. This approach helps harmonise terminology and supports cross-study comparisons (48). Below, we outline selected strategies, organised according to Leeman's classification system (49):

**Capacity-building strategies**: Following the internal pilot, mandatory preparatory workshops with municipal management representatives were implemented. These sessions, led by the principal investigator (PI), aimed to introduce the ELR, clarify roles, and build implementation readiness. Detailed roll-out plans with timelines were also distributed. To support knowledge building and adoption, pre-recorded videos (*n* = 10) and written resources were shared with managers, occupational therapists, and housing staff. Before the trial started, managers were responsible for facilitating staff viewing of these web-based educational videos during work hours. To maintain fidelity and address challenges, occupational therapists also received regular in-group coaching throughout the trial.
**Integration strategies**: Occupational therapists facilitated weekly meetings with residents and developed written recovery plans to support resident goals and activities. The recovery plans were used to monitor clinical processes by mapping activities, strategies, and goal progression. To enhance intersectoral collaboration and team-based care, the occupational therapist's role was redefined to enable closer coordination with housing staff and residents. Rather than staff assessing needs and contacting occupational therapists when needed, the occupational therapist proactively engages residents in outreach-based rehabilitation. In addition, housing managers led monthly reflection sessions to promote collaborative learning and address feasibility challenges among housing staff. Pre-written reflection questions for these sessions were distributed in advance.

### 2.2 Context description

The current study explores housing staff discourses regarding their experiences of implementing a novel intervention within the context of an RCT. In the trial, 16 municipalities participated with a total of 60 housing units. To be eligible, units had to provide services to individuals with SMI, excluding units for those with intellectual disabilities, and have sufficient access to an occupational therapist. Most of them corresponded to a type-1 facility in the STAX-SA taxonomy and provided 24/7 access to on-site staff. The usual practices at these units include a wide range of residential services and psychosocial support provided by housing staff, which vary depending on individual approaches, staff commitment, and unit norms. Managers are typically based off-site with substantial workloads, which places considerable responsibility on housing staff to manage their daily practices (10). Trial process data indicate that up to half of the managers did not fully adhere to the intervention, leading to information not being communicated to housing staff on time. Collaboration between occupational therapists and housing staff also tends to be challenging, with rehabilitation efforts limited to short-term, sporadic interventions, such as the prescription of technical aids (50, 51).

### 2.3 Data collection

Seven focus group interviews were conducted with housing staff (*n* = 23) from selected units. Frontline practitioners are considered good interview subjects when exploring barriers and unintended consequences, as they often are left responsible for “picking up the pieces” after an implementation process (46). A semi-structured interview guide was developed using the CFIR (Consolidated Framework for Implementation Research) web application (52). The framework includes 48 constructs and is organised into five domains: innovation, outer setting, inner setting, individuals, and implementation process (53). Designed to identify barriers and facilitators to implementation effectiveness, CFIR ensures that the interviews capture key constructs relevant to understanding implementation outcomes (54). The interview guide was further refined using Brönnimann's framework for developing realist-inspired interview questions (55).

The focus group interviews began with introductory questions aimed at understanding the organisational context. Staff were asked to describe their role, their unit, the number of residents participating in the intervention, their onboarding procedures, and their relationship with their manager. Selected CFIR constructs were then explored, including, for example, Tension for Change (“Do you perceive a need for ELR or similar interventions at your unit?”), Self-efficacy (“To what extent did you feel capable of working according to ELR?”), Patient Needs and Resources (“Did you encounter any barriers that limited residents' participation in the intervention?”), Intervention Characteristics (“Can you describe a typical situation in which you worked with residents using ELR?”), Leadership Engagement and Relative Priority (“How important is management support and engagement for the way you work?”), and Compatibility (“How well does ELR fit with your organisation and existing work practices?”).

The focus group interviews were conducted by two PhD candidates (AS and MH). AS conducted five interviews, and MH conducted two. Participants were informed that the interviewer was a member of the research team, but they were still encouraged to provide both positive and negative perspectives. The focus groups were conducted at the supported accommodation facilities during working hours, often in conjunction with regular staff meetings. Neither housing managers nor occupational therapists were present during the interviews. Each focus group was conducted on a single occasion, with the aim of maintaining consistency across interviews while promoting diverse perspectives by encouraging disagreement and asking challenging questions (56). Previous interviews were used to enrich forthcoming iterations of the interview guide. Participants were not informed of which sample group (smooth or problematic) their unit belonged to, and the format was standardised across both groups. The topic of the interviews was presented as staff experiences of the implementation of ELR in their own unit. All interviews were audio-recorded, and brief field notes were taken during the sessions. Transcripts were not returned to participants for comments or corrections.

### 2.4 Data analysis

The material collected during the focus groups was analysed using CDA (40). CDA enables the exploration of how organisational change is mediated by discursive strategies and how representations are negotiated, reproduced, and contested within specific social contexts (38). The social problem explored in this study is how staff respond to, negotiate, and resist ELR in relation to established practices, which may constrain resident's opportunities for activity engagement and self-determination. In this study, we adopted Norman Fairclough's dialectical–relational approach and analysed the corpus using his three-dimensional analytical framework, in which discourses are conceptualised simultaneously as a text, discursive practice, and social practice (57). Events, experiences, and reflections expressed by staff were treated as discursive and analysed through this framework (see Table 1).

**Table 1:** Overview of the analytical process.

Stage	Dimension	Description of our analytical process
Description	Text	Examining how staff describe implementation issues through their language use in the discourses
Interpretation	Discursive practice	Exploring how staff construct, negotiate, and make sense of implementation issues in the discourses
Explanation	Social practice	Seeking to explain how organizational and social context shape the implementation issues identified in the discourses applied by housing staff

Interviews create a controlled, co-constructed discursive setting shaped by both the interviewer and participants. Consequently, analysis of discursive practices is limited to this context and does not necessarily represent naturally occurring interactions. Textual analysis and discursive practice analysis were therefore merged in the findings. The role of the analyst in CDA is to critically examine the relationships between language use and social practices, recognising that language functions as both a means of communication and an instrument of power (58). While actors are ideologically positioned, they retain agency through personal and social cognition, including memories and opinions (59). What is perceived as “common sense” therefore emerges through engagement in particular social contexts, becoming a form of habit (58). In this study, discourse is understood as a relatively stable representation of reality, meaning multiple discourses can coexist (60).

Interviews were transcribed verbatim using a form of denaturalised transcription, emphasising content over linguistic features such as pronunciation or vocalisation (61). The transcribed material represents our corpus. Both interviews and analysis were conducted in Swedish to preserve linguistic and contextual nuances, with the material being translated into English only for publication. The analysis began with all three analysts independently analysing two focus group interviews. This was followed by an analytic meeting to reflect on and discuss conflicting interpretations, thereby refining and deepening our understanding of the corpus. The remaining interviews were then distributed among the research team. AS analysed all interviews, while ML and LRS each analysed a subset and contributed alternative interpretations to refine and strengthen the final analysis.

An inductive coding approach was used (62), whereby excerpts were assigned multiple, non-exclusive colour codes (see Table 2). These codes were intended to facilitate the identification and highlighting of key tensions by drawing attention to discursive patterns, power relations, conflicts, and emotional content within the corpus (63). Excerpts were selected to identify discursive patterns and were systematically compared with the full corpus, paying special attention to deviant and contradictory cases. Excerpts were then abstracted into discursive representations through an interpretive analysis. These discursive representations should not be understood as top-level categories; rather, they function as analytical constructs that capture distinct combinations of discourses and contextual challenges. All discursive representations may include both resistance and adaptation, with implementation issues manifesting differently across units.

**Table 2:** Examples of analytical colour codes.

Codes	Description
Green	Discursive practices/language use in practice
Dark green	Interactions between discourse and social, organisational, or societal structures
Blue	Discourses of normality, routine, and the taken-for-granted
Orange	Discourses of novelty, change, and innovation
Brown	Power relations and authority
Red	Conflict, tension, and contestation
Dark red	Discursive contradictions, deviations, and counter-narratives

#### 2.4.1 Positioning and reflexivity

All authors contributed with distinct analytical perspectives in this study. ML, the developer of ELR and principal investigator of the RCT, contributed in-depth knowledge of ELR and the social psychiatry context, collaborative recovery and rehabilitation approaches, and extensive experience in intervention and implementation research, as well as qualitative methods. ML was not familiar with the specific housing units or the staff; however, her close involvement in the project influenced her understanding of the implementation issues.

The corresponding author, AS, was a PhD candidate. Although involved in the blinded analysis of the RCT, he was not involved in the development of ELR or the design of the trial. AS contributed prior experience as a social worker and housing manager in supported accommodation. He also met many of the staff participating in the interviews, which may have enriched his interpretation of the data and context.

The final analyst was LRS, an experienced psychologist and implementation researcher, who had no prior involvement in the RCT or intervention development. In addition to her expertise in implementation research, her relative distance from the project provides a less-embedded perspective on the data, thereby fostering analytical reflexivity and critical discussions within the research team.

## 3 Findings

In the corpus, we identified five main discursive representations of staff's engagement with the intervention. Within these representations, we observed both similarities and differences in how they discussed the intervention, the residents, the collaboration, and their own organisation. Staff within the same unit often shared perspectives, but variations were common between units. Identified discourses may therefore encompass multiple, and potentially conflicting messages. The underlying context remains consistent across all discursive representations, so while the focus in each may differ, they emerge from and reflect the same organisational reality. All excerpts and quotes are italicised and pseudonymised.

The analysed corpus consisted of seven focus group interviews with units across four municipalities (see Table 3). These units represent all trial waves, spanning between 2021 and 2024, with two units receiving a delayed intervention as part of the waitlist control design. Between two and five staff members participated in each interview. Two of the interviews were conducted via web meetings, while the remaining interviews took place on-site. On average, the focus group interviews lasted approximately 46 min, with one notable exception in which the session was shorter than the others.

**Table 3:** Description of participating units.

Description
Focus group 0103: Five housing staff from municipality 1, which was part of wave one in the RCT. Interviews were conducted in person, with 40:41 min of recording, and recruited three residents, of whom all completed the trial
Focus group 0104: Four housing staff from municipality 1, which was part of wave one in the RCT. Interviews were conducted in person, with 48:28 min of recording, and recruited one resident who also completed the trial.
Focus group 0105: Two housing staff from municipality 1, which was part of wave one in the RCT. Interviews were performed via a digital meeting, with 25:17 min of recording. They were allocated to the waitlist and recruited four residents, of whom one completed the trial.
Focus group 0201: Three housing staff from municipality 2, which was part of wave three in the RCT. Interviews were conducted in person, with 49:27 min of recording, and recruited three residents, who all completed the trial.
Focus group 0301: Five housing staff from municipality 3, which was part of wave four in the RCT. Interviews were conducted via a digital meeting, with 41:25 min of recording, and recruited two residents, who all completed the trial.
Focus group 0401: Two housing staff from municipality 4, which was part of wave two in the RCT. Interviews were conducted in person, with 62:43 min of recording, and recruited four residents, of whom two completed the trial.

### 3.1 Diminishing novelty of ELR and legitimising familiar practices

It was common for staff to frame ELR as a natural extension of the current organisation. By conflating the intervention's components with their usual practices, they effectively diminished the intervention’s perceived novelty. This framing signals alignment between the intervention and organisational goals but also limits the intervention's disruptive potential.

Excerpt: Interview 0401

Interviewer: And what did you feel then? Now you are supposed to start with a new method.

Speaker 1: But it wasn't that novel when you started to read. Like, we do a lot of this already.

Speaker 2: Yes, exactly, I think so too. You do a lot for them in everyday life that way. Yeah, it was no problem. It is really about getting the residents involved. That's the thing. They're the ones who are more affected when something new is introduced, you know. But I think it has been really good, and it turned out well, we got to learn how to crochet. Those of us who couldn't.

Speaker 1: Because there were a lot of us that couldn't

Speaker 2: Yes exactly. Snapchat, I've started doing that now afterwards. There is like a lot of stuff that exists, that you need to learn, even us regular folks, you know, that don't live at a supported accommodation.

Interviewer: Exactly

Speaker 2: Just the fact that we had the occupational therapist come in and contribute, that was really lovely! It was great to discuss different things, and they [residents] got another person to connect with, someone consistent they could turn to. It added something fresh to everyday life.

Interviewer: So, it was not so much that you felt that “what is this?” when you were introduced to the model, it was quite easy to grasp?

Speaker 2: No. I think it really is a modernised version of what's always been done, actually.

Speaker 1: And you get like something to work according to, before you just do things as they came up in everyday life. But now you've got something more concrete, “this is what we're working according to”. It is a bit clearer. Something to latch onto.

Speaker 2: Exactly, it is like that.

#### 3.1.1 Text and discursive practice

This excerpt illustrates how staff, discursively, seems to align the intervention with their established practices, thereby reducing its perceived novelty. The phrase, “…it is like a modernised variant of what we always have done,” exemplifies this tendency to conflate the intervention with existing practices. This phrase positions the intervention not as a disruption but as a natural development of the whole organisation. This type of rhetoric signals alignment between ELR and the organisation's mission but can also be interpreted as a way for staff to reaffirm the value of existing practices. By using the term “a modernised variant” as an analogy, the intervention is positioned as superior while also framing it as fundamentally the same, which would presuppose that established practices function quite well.

Staff emphasise that ELR generated positive changes in attitudes, while simultaneously diminishing its influence on everyday practice: “Even if (…) the methods themselves may not be that different, the mindset has (…) received a big boost.” Through hedging (“may not be that different”), the respondent frames the intervention as a modest shift from established practices, while still recognising its capacity to generate organisational change. In other units, the intervention is believed to have legitimised specific practices in the organisation: “it also creates a lot of ideas, and like, this with approval to do. To go aside with this person that is involved in like ELR (0401).” This notion of receiving approval or permission underscores the importance of organisational culture in implementing and sustaining new practices. Since the trial provided few additional resources, this sense of permission must have emerged from perceived interpersonal approval within the organisation. In many units, receiving this approval to just be with the resident is framed as a key facilitator for successfully implementing the intervention.

Even in units where implementation had been more challenging, staff tended to conflate ELR with existing practices. For example, one respondent described how he thought that the intervention replicated how their existing service delivery plans were intended to function: “The concept is the same as the service delivery plans… We were thinking it [ELR] would be service delivery plan plus, with extra resources. (…) the concept is familiar, and in the best of worlds this is what the service delivery plans would be (0104).” This statement can be understood as both a subtle critique of existing service delivery plans, which are portrayed as not functioning as intended, and as praise of ELR, which is framed as a means of strengthening existing practices. However, the tentative phrasing (“we were thinking it would be”) suggests that these expectations were not fully realised in practice. The intervention was also often mixed up or merged with other similar approaches found in this setting, such as person-centred care: “I can recognise the method, even if it is called something else (0402).” Our interpretation is that staff are unable to clearly distinguish ELR from other approaches, which could create uncertainty about what is novel about the intervention, the reasons for its introduction, and the changes expected of them.

In general, staff found the intervention valuable, but they often thought it was difficult to apply in their everyday practices. This tension was apparent in discussions about the educational material: “It just felt so complicated! Even though it doesn't really seem like it should have to be that complicated. But maybe we're not all that ‘educated’ either (0104).” This statement conveys frustration and emotional intensity toward what is perceived as unnecessary complexity. The statement “it doesn't have to be that complicated” contains a normative judgment about how the process ought to be, while the description of themselves as “not all that educated” functions as a form of self-deprecating hedging that mitigates the preceding criticism. Furthermore, the use of the pronoun “we” constructs a collective identity, transforming individual frustration into a shared experience. On the other hand, some respondents expressed that the educational material had sparked interest in the project: “That's when you got your expectations up! (0201).” Notably, staff rarely referenced key theoretical concepts, such as personal recovery and CHIME, when discussing the intervention or educational material.

#### 3.1.2 Social practice

One interpretation is that conflating the novel intervention with established practice enables staff to align themselves with ELR's underlying values while resisting the organisational changes associated with its implementation. This could be understood as both a protective strategy and an example of biased assimilation (64). By claiming that they already do this work, staff construct established practices as adequate, rendering change redundant, while also avoiding admission that previous approaches were inadequate. Staff who have strong preexisting assumptions about their own practices might focus on surface-level similarities between ELR and their usual practices, while overlooking substantive differences. Another interpretation is that this is a way for staff to “domesticate” the intervention by aligning it with local practices and values, thereby legitimising specific ways of working, and making the intervention more acceptable to colleagues.

There is a risk that this conflation could neutralise some of the intervention's disruptive potential and contribute to a form of illusory consensus, whereby staff perceive themselves as adhering to the intervention while they are not. It is important to raise the question: if staff adhered to all components of the intervention, would it not be perceived as a larger disruption than is reflected in the focus groups? Our interpretation is that several interventional components were likely overlooked during implementation. If staff lacks knowledge of the intervention, they would be unable to distinguish it and would therefore conflate it with other approaches. This is also supported by process data from the trial, which showed declining engagement with the educational material over time, that meetings organised by the managers were not conducted as planned, and, most tellingly, that staff rarely referenced the theoretical concepts central to the intervention.

### 3.2 Restricting resident potential in response to organisational norms

All units claimed that they are, at least in theory, positive about the intervention. Still, their own residents were often framed as having too complex disabilities to benefit from it. These discourses suggest a tension in perceptions of the potential of residents, as well as a paternalistic discourse about what type of activity and for whom activity engagement is valuable. Staff expressed a need to constantly mediate between practical constraints and the demands of the intervention, leading in the promise of resident self-determination being perceived as a direct threat to organisational stability.

Excerpt: Interview 0103

Speaker 1: The idea is great! But it was too complicated. Our residents have problems with… When we introduced this, it was like a lot of information, which they cannot handle.

Speaker 2: And we just couldn't handle it. I mean, we couldn't cope, with the language… with the structure, I mean, it was just all lined up.

Speaker 1: Yes, it was difficult.

Speaker 3: Yeah, it was way over their heads…

Speaker 1: And then I don't know if we had the wrong occupational therapist, but she… she set the bar too high for our residents when we started this for real.

Speaker 2: Because ours are really at quite a low level, and in that case, you maybe need to put it more at… more of a children's level, that's what we would have needed. Not an adult or professor's level. University level…

Interviewer: Okey, what… Could you give some examples of such activities that were brought up?

Speaker 1: Well, the activities themselves weren't difficult. One was going out for coffee, one was practicing taking the bus, and one was cooking once a week. Sausage and macaroni. So, it wasn't anything advanced, but it got blown up into this huge thing. And they can't handle that.

#### 3.2.1 Text and discursive practice

In this excerpt, staff express dissatisfaction with both the occupational therapist and the complexity of the informational material. They characterised the intervention as too demanding for residents, who they argued needed information simplified to “the level of a child.” By depicting residents as children, staff legitimise their own authority to determine what residents can understand and do. In addition, by positioning themselves as necessary interpreters (“they cannot handle”), staff suggest they must mediate what information residents receive. The contrast between “child” and “professors” also functions as an implicit critique of the project, suggesting that the academically developed material was not adapted to resident's actual capabilities.

Respondents who critiqued the intervention's complexity often emphasised the perceived dissonance between the intervention's target population and their own residents, who they argued had particularly severe disabilities or were especially hard to motivate: “S1: I think this would have suited better in a place where there are… more high functioning [residents]. S2: Yes, high-functioning, people who want something (…) Here, though, they don't have that drive! (0103).” By arguing that the intervention is only appropriate for “high functioning” residents, who are described as not residing at their unit, staff implicitly label their own residents as low functioning. One interpretation is that this binary framing functions rhetorically to shift responsibility away from the organisation and onto residents, positioning their motivation and capabilities as the primary determinant of the intervention's success.

It was not uncommon for staff's frustration with the perceived lack of progress to manifest in pessimistic assessments of residents' capacity for change: “… how long ago was this? He [resident] still can't boil pasta or fry sausage [laughs]. And we do that once a week, but it doesn't make any difference (0103).” In this statement, residents' progress is teased, arguing that despite staff's sufficient efforts, the resident still has not learned to cook. We observe how agency is discursively constructed using different verbs, with staff positioned as active and capable agents (“we do that once a week”) and residents positioned as passive and lacking incapable (“He still can't boil pasta”). One interpretation is that constructing residents as hopeless cases serves a protective function for staff's professional identity. Within this framing, implementation failures or limited progress are attributed to resident's inability to change, rather than to shortcomings in the services.

It was not only residents' progress that was criticised but also the type and scope of the activities they chose to engage in: “we saw other areas which she needs more in her life. Sure, she should decide by herself. That is important. But you might have a diagnosis which makes it so that you can't see all the parts that you would need to train on (0104).” Although the value of self-determination is explicitly acknowledged, expressions such as “Sure” and “That is important” can be interpreted as performative signals of alignment with dominant discourses of autonomy and person-centredness. By following the endorsement of self-determination with the conjunction “but,” the respondent discursively pivots, once again positioning clinical expertise as taking precedence over the resident's right to autonomy. In addition, this implies staff can identify resident's true needs, while residents are constructed as largely unable to recognise those needs themselves.

Many activities were framed as too trivial or already part of usual practice, as one respondent stated, “… it wasn't anything advanced, but it got blown up into this huge thing (0103).” This statement effectively constructs the intervention as not being disruptive enough to warrant the attention it received. The metaphor of an explosion underscores the excessiveness of the approach. One interpretation is that this statement functions as a counter-discourse to contest the perceived dominant discourse through which occupational therapists and residents describe the intervention. Another respondent remarked how they had “…actually already started up earlier (0301)” when referring to a planned activity, insinuating that the occupational therapist was redundant. An even more explicit example of this is how one respondent replied: “Well, we didn't really need an occupational therapist to figure that out (0201).”

Although staff in pessimistic units often dismissed the activities as trivial, they frequently used hyperbole descriptions of resource-intensive activities, such as trips and large events, to amplify the perceived risks of allowing residents to choose activities freely: “… about them being able to choose pretty much themselves. Well, with what? We don't have any cars! We cannot take them anywhere (…) the ambition has exceeded what we are able to offer (0104).” In this statement, self-determination is framed as a potential threat to organisational stability due to logistical constraints within the organisation. The use of a rhetorical question (“Well, with what?”) emphasises staff's frustration with what is requested from them. By framing the intervention's ambitions as unrealistic, staff implicitly criticise the intervention's design and attribute implementational challenges to a misalignment between interventional demands and available resources.

Many respondents still described some form of need for change: “I mean, the whole point of us being here is because we believe that the residents should have a good life! That they should have a good everyday experience. But instead, we just end up doing the bare minimum every evening, and it is depressing (0104).” The term “bare minimum” implies that the respondent recognise that the organisations have potential to deliver higher quality services, but that they feel constrained by limited resources. The use of “should” signals a normative stance, reflecting a moral commitment to delivering better care. This could be interpreted as emotional frustration over the perceived tension between their ideals and the reality of their everyday work.

It was not uncommon for staff to position themselves as being responsible for balancing resident's needs with scarce resources: “…we also must think about the economy and everything else, like staffing. (…) We cannot go fishing for a whole day (0103).” This tension between ideals and available resources was also apparent in staff's reflections on the intervention's feasibility. Staff argued that ELR was only feasible if the number of participants remained limited: “We were lucky that not so many wanted to participate. … Fitting in even more would probably have been tight (0105).” By framing the limited uptake as a matter of luck, the respondent implies a lack of control over both resources and implementation conditions. The statement further positions resource scarcity as a key determinant of what can realistically be offered by suggesting that the unit was already operating near capacity.

#### 3.2.2 Social practice

Our pre-understanding of this context is that it is often characterised by a scarcity of time, staff, and leadership. In the corpus, it seems like staff use contradictory rhetorical strategies to manage the tension between ideals and organisational demands. Staff simultaneously trivialised the intervention, suggesting that it was not disruptive enough to warrant change, while exaggerating hypothetical scenarios to justify gatekeeping and risk aversion. Our interpretation is that these contradictions are reinforced by a paternalistic discourse in which residents are constructed as lacking both decision-making capacity and motivation. Within this discourse, gatekeeping is positioned as a necessary and legitimate response to resident's perceived limitations.

Another interpretation is that these rhetorical strategies reflect a mode of reasoning grounded in an institutional discourse of efficiency and resource management. Rather than prioritising resident's wishes and needs, staff frequently frame the intervention in terms of its implications for organisational stability and resource availability. It is important to acknowledge that scarce resources are likely a genuine concern for staff, shaping the conditions under which they provide services. One possible explanation is that, in the absence of leadership, staff are compelled to adopt managerial rationalities to navigate tensions arising from resource scarcity (see the Blurred accountability, low expectations, and inevitable constraints section). As staff have limited influence over available resources, tensions may generate frustration and risk-aversion, contributing to the framing of resident's needs as a resource management problem.

### 3.3 Guarding the boundary between insiders and outsiders

Staff describe their professional role as characterised by closeness to the residents. While this familiarity was often regarded as advantageous, it was also perceived as making it difficult for them to motivate residents, as the relationship had often become stagnant. Consequently, staff expressed a need for support from external actors from the outside. At the same time, the involvement of external actors could foster a sense of rivalry, particularly in an organisation built on cohesion, where the “outsider” holds decision-making authority.

Excerpt: Interview 0201

Speaker 3: And I would think that they [residents] would feel good if people from the outside came as well.

Speaker 2: Yeah

Speaker 3: I do, because we end up like mothers in the end. We kind of go around nagging. There are quite a lot of guys that live here. Young guys. It would be fun if there was a guy who, well…

Speaker 2: That came with some ideas and motivation! Speaker 3: Yes, and since they're also not allowed to have a contact person, they hardly meet anyone their own age. It is really just them and us, in a way.

Interviewer: Right

Speaker 2: It is always easier to, you know, say no to us than to someone coming from the outside. (laughs a little) “No, I don't want to go today” (Imitation).

Interviewer: What do you find most difficult when you try to motivate them? You've touched on it a little, but if you could elaborate.

Speaker 2: Their own will (laughs), I was about to say.

Speaker 3: It is their own will.

Speaker 2: Yes.

Speaker 3: You're almost prepared for them to say no (laughs). We've been working for so long and we have not progressed. We don't get any new inspiring ideas either. Like we only have each other to brainstorm with. And it is hard to pull them away from the computer or motivate them to do anything. Then you lose motivation yourself, when you don't know what to do…

Interviewer: No (Confirmative)

Speaker 2: It is easier to tell us: “No, today I'm not doing it” (Imitation). They feel that secure with us. It is mostly us. It is like you say… you end up becoming a bit of a backup mom.

#### 3.3.1 Text and discursive practice

In this excerpt, staff articulate a desire for collaboration with external actors, referring to those “from the outside,” meaning persons who do not work within the unit. Differences in age, gender, and roles are used to rationalise why staff find it harder time to motivate residents than external actors do. Here, they use the metaphor of a parent to describe their own role, signalling a sense of both responsibility and authority. This also represents a transgressive blurring of institutional boundaries, where parental and professional identities overlap. By applying this metaphor, staff construct a discourse of a long-term, stagnated relationship, in which they have exhausted most of their motivational strategies. Consequently, when attempting to engage the residents, they are positioned as adversaries, where they must resort to “nagging” to enforce their will. It is then argued that an external actor, not yet affected by this stagnation, would find it easier to engage residents in activities.

Staff argue that this long-term, stagnated relationship also makes them lose motivation: “We don't get any new inspiring ideas either. Like we only have each other to brainstorm with. (…) Then you lose motivation yourself, when you don't know what to do.” Staff's reliance on each other and the absence of “new inspiring ideas” foster a discourse of isolation. By implying a lack of external stimulation and support, they can explain both their personal demotivation and a sense of directionlessness. When asked what the intervention lacks, one respondent replied: “Well, but maybe that a third party would step in… because it is harder for us to… it felt like everything dependent on us (0105).” The critique is grounded in a perceived imbalance of responsibility and lack of organisational support, with staff portraying themselves as having assumed primary responsibility for the intervention. Their use of soft hedging (“Well, but maybe”) allows them to cautiously express a desire for more external support while mitigating the force of their criticism.

In many cases, the occupational therapist was able to fill the role of this third party: “I thought it was really comforting to have her to lean on and reflect with. We haven't had that before, this kind of closeness (0401),” which frames the increased involvement of the occupational therapist as something novel and positive. The occupational therapist was often positioned as part of the staff's referral network, rather than as a colleague, establishing a physical and social distance between the two professions: “They [occupational therapists] are stationed somewhere else (…) this was the tricky part, with communication and scheduling appointments. They can't have those spontaneous conversations with the participants [residents] in the same way we [staff] can (0301).” Here, different pronouns are used to create a distinction between being an insider and an outsider. By emphasising that occupational therapists are unable to engage in spontaneous conversations, they also assert their unique relational and proximal connection to the residents.

If occupational therapists intervened on behalf of residents without having established relational legitimacy, their actions could be perceived as antagonistic: “Like, we have known her [resident] for a long time. Then she [occupational therapist] started to school us on how ADHD works (0104).” In this argument, the respondent constructs a relational connection to the residents as a source of legitimacy. When the occupational therapist then attempted to exercise authority without establishing such a connection, their actions were perceived as condescending. Two notable exceptions were identified, both occurring in separate units in which the occupational therapist became incorporated into the inner setting: “Then she kind of became part of the group here too, because all of a sudden she would just show up, you know (0401).” Staff described a reduced relational distance, with the occupational therapist's presence and tendency to simply “show up” at the unit facilitating collaboration and fostering a sense of inclusion within the inner setting.

Our interpretation is that the organisations function primarily as a cohesion-driven system, in which shared values, work styles, and attitudes are believed to be virtuous. Staff often described ethos and character as the main criteria's for assessing the value of colleagues. For example, when asked about the most important aspect for new employees, one respondent answered: “… ensuring that we are on the same page, as much as possible. … So, we are quite careful about who is allowed to come in as a substitute (0201).” The phrase “being on the same page” discursively signals a desire for consensus and shared understanding, while simultaneously positioning staff as gatekeepers who determine who is allowed to work as a substitute. This can serve as a mechanism for maintaining organisational cohesiveness.

Several units described that they had learned the importance of cohesiveness from past incidents: “We've fallen into those traps before; we know that doing our own thing often turns out badly. It can go really badly (0104).” This implies that the organisational culture has developed over time and could also serve to establish a sense of tradition. Staff often emphasised the uniqueness of their own unit, both for residents and for staff. For example, when asked about the working culture at the unit, they answered: “S1: I think we care more about each other. Maybe because we are so few, and we work so long hours, so we are kind of forced to hang out, and get to know each other. S2: That's what people say too, those who came from other units, here we are so nice! (0103).” Here, the group distinguishes itself from what they perceive as an “average” unit, by highlighting how their working environment is much nicer.

Even when managers had little involvement in the daily services, when asked about it, staff almost always reaffirmed the manager's local knowledge: “Well, not in the daily no. (…) [but] she does have knowledge, I would say, about all the tenants and the issues and such. So, she is familiar with it (0301).” By emphasising the manager's familiarity with the services and residents, staff appear to grant them relational legitimacy, positioning them as an insider within the inner setting. When asked about their manager, another responded said, “… she was one of us on the floor. So, I think it is quite natural for her. (…) Like it becomes more allowing when you have a manager who knows what you need, what I need to do for the residents (0401).” The use of the pronoun “us” functions to create in-group solidarity, discursively erasing the hierarchical boundaries between them and the manager. At the same time, it establishes a shared collective identity that implicitly excludes outsiders. Having been “on the floor” grounds the manager's legitimacy in shared experiences, rather than professional qualifications. One interpretation is that this identification reinforces a sense of sameness, making it easier for staff to relate to the manager while subtly constructing a culture that values relationships and practical knowledge.

#### 3.3.2 Social practice

There seems to be a tension for change within the organisation, and staff frequently request support from external actors, believing that they could break their perceived stagnation in the staff–resident relationship. Our interpretation is that this stagnation fosters a discourse of hopelessness, in which staff constantly doubt their capacity to motivate residents. Since these services rarely receive targeted improvements, this hopelessness risks becoming materially reinforced, causing organisational constraints to be feel permanent and unchangeable. Nevertheless, the organisation's cohesive culture makes it difficult for outsiders to gain acceptance, requiring new actors to establish relationships with both staff and residents to obtain legitimacy. Within the organisation, this criterion of relational legitimacy appears to be dominant, and different labels are used to establish the boundary between insiders and outsiders. Although staff acknowledge that external actors could address their perceived stagnation, they resist relinquishing control to those lacking relational legitimacy (see Intersectoral collaboration and issues of ownership and power).

Another interpretation is that staff seem to have strong loyalty to the inner setting of the organisation, which is constructed around shared experiences and perceived relatability. As these services are often rendered invisible in public discourse, staff must protect their collective interests themselves. However, this also serves as a gatekeeping mechanism, making the introduction of new perspectives challenging. How occupational therapists are perceived in these units seems to depend a lot on how well they align with the local culture. When accepted, they are seen as valuable colleagues; when not, they are portrayed as fleeting visitors with a limited understanding of residents' inner lives. In contrast, managers who are very distant from the floor retain insider status, perhaps because they are perceived as loyal to the unit. Yet why is the occupational therapist still considered an outsider? While time and familiarity likely play a role, one possible explanation concerns institutional allegiance. Occupational therapists operate within municipal healthcare teams, which are accountable to different management structures, legal frameworks, and professional mandates than housing staff. This could make them not only strangers but also institutional outsiders, rendering them more vulnerable to criticism and resistance.

### 3.4 Intersectoral collaboration and issues of ownership and power

Staff often positioned the occupational therapist as having greater authority and responsibility for the implementation process, while emphasising their own close relationship with residents. This discursive positioning may be interpreted as reflecting a hierarchical relationship, which in some cases appeared to contribute to conflict. Furthermore, staff's tendency to attribute implementation challenges to external constraints or the actions of others may suggest a limited sense of ownership of the implementation process.

Excerpt: Interview 0103

Speaker 2: Maybe we would have needed an occupational therapist who already knew, well, us and the residents beforehand, I think.

Interviewer: Right, so you had a new occupational therapist.

Speaker 1: Yes, I think they swapped with each other, to not be in their own area.

Speaker 2: Although it was bad for us.

Interviewer: Right, how did you work with the occupational therapist before the project? How often where they are and how much contact did you have with them then?

Speaker 2: I mean, that's where it got crazy because we actually had X (the old occupational therapist), but then we got someone named Y (the new occupational therapist). And it turned out wrong; we didn't have any relationship with her…

Speaker 3: No, I think it would have been better if we had the one who was our regular.

Speaker 1: She knew the residents as well. She has met most of them, so she knows what level they are at.

Speaker 2: It felt like we had to sit and explain. We spent a lot of time explaining to the occupational therapist, like, “that's not how it works”. It was like we were wasting time on it, somehow.

#### 3.4.1 Text and discursive practice

In this excerpt, staff express dissatisfaction with the occupational therapist appointed to their unit during the trial. The dialogue is marked by frustration and a sense of inefficiency, as evidenced by phrases such as “it turned out wrong” and “we had to sit and explain.” These expressions create a perceived disconnect between the occupational therapist and staff, which is attributed to the absence of prior familiarity, trust, and shared understanding. By repeatedly referencing the difference between this new occupational therapist and their predecessor, staff reinforce the importance of established relationships as a source of legitimacy and effective collaboration. Staff believe that having a pre-understanding (“she knows what level they are at”) of residents is essential to communicating with them effectively. Using statements like “she knew the residents as well” and “who already knew us and the residents,” staff establish that familiarity is often valued more highly than formal competence. The framing of the situation as being “crazy” and “wasting time” conveys frustration with the intervention and may reflect a perception that it failed to account for the importance of contextual knowledge.

In addition, the formal authority of the occupational therapist seemed to make it difficult for staff to feel involved in the process: “It feels like we weren't part of this at all. So, I don't know, we might get critique for that, but (…) it didn't feel like we were invited (0201).” When asked if they knew what the intervention was about, one unit answered: “S1: Both yes and no. It was like the occupational therapist who was running it. S2: We became more like an extended arm, which can be difficult, because you don't really know what your actual role is (0301).” The hedging of “both yes and no” suggests confusion of their role in the intervention, while the metaphor of “an extended arm” is used to convey a lack of agency and a sense of being overlooked.

Our interpretation is that this type of phrasing underscores the staff's frustration with being sidelined in the decision-making and their struggle to define their role within the intervention. Another unit described how lack of information diminished their sense of ownership, contributing to feelings of exclusion: “We were just the implementers. We never found out if anything actually went anywhere (0201).” Staff felt that they lacked communication with the occupational therapist and expressed that information often had to be relayed through residents, which was argued to increase the risks of misunderstanding given resident's disabilities: “In the end we almost didn't know who wanted this, for whom we are doing this (0104).” Here, staff implies that the activities did not originate with the residents but rather reflected the will of the occupational therapist.

On the other hand, when tensions emerged, residents were not described as passive but rather as active participants in the conflict: “One used the occupational therapist to talk about how bad we were (0103),” arguing that communication between the residents and the occupational therapist created an unfair image of the staff. In some units, the occupational therapist was seen as destabilising established power dynamics within the staff–resident relationship. This is illustrated by one unit's description of being portrayed as “some type of fascists that tried to control her [resident] (0104).” Another example is how staff, as gatekeepers of shared resources, struggled when residents attempted to assert their right to activities: “But you could really get your head bitten off for that, seriously, because it was damn well her [resident] right to have that. Even if it clashed with ten other things, she was going to have that time (0104).” In this story, the resident challenged staff's authority, where the phrase “damn well her right” portrays a strong, almost defiant stance. The individual is believed to place their own needs as non-negotiable, without regard for conflicting priorities (“even if it clashed with ten other things”).

Even more successful units expressed tensions when resident's wishes conflicted with organisational priorities. For example, one unit described how they dealt with a resident who spontaneously wanted to go to the store: “Then it is like, well, we just drop everything… Can you [colleague] stay in there, because now I must go (0402).” Here, the tension is not framed as a conflict but rather as an obstacle that staff must overcome. The phrase “we just drop everything” signals emphasis on flexibility and adaptability. The conversational tone and the informal delegation to a colleague further highlight the spontaneity and collaborative nature of the staff's response. This is important, as flexibility was frequently raised as a key competence in the organisation: “… you learn to. (…) be flexible, maybe the matter you had in mind when you went there doesn't turn out that way, because today is a different day (0201).”

When asked about the potential benefits of collaboration, assuming less friction and conflict with the occupational therapist, one respondent answered: “I think it would be great. The occupational therapists are the ones in the health care team that we have the least contact with. (…) I wouldn't mind at all to have more contact with them in our daily work. (…) When it comes to the goals (…) we were not that involved. [But] the methods used were good. (0104).” This response softens the previous critique and reinforces the occupational therapist's professional expertise. Our interpretation is that staff's frustration stems not from the intervention's methods themselves but rather from issues related to communication, relationships, and resource allocation.

In units where staff perceived themselves as having primary responsibility for the intervention, we observed expressions of abandonment: “I had hoped, I really expected, that there would be closer collaboration between us and her [occupational therapist]. I truly did expect that (0201).” In other units, staff expressed a sense of loss following the trial, underscoring the value of the occupational therapist's contributions: “… she [occupational therapist] managed to bring out things that we hadn't been able to reach. She found different ways. (…) She won't have as much time here now, and that is a shame, because it is needed (0402).” The phrase “she managed to bring out things that we hadn't been able to reach” frames the occupational therapist as an active agent who facilitated access to experiences that previously were inaccessible to staff.

#### 3.4.2 Social practice

Traditionally, in this context, staff are responsible for assessing the needs of residents and contacting the occupational therapist when required. The intervention reverses this process, positioning the occupational therapist as proactive and engaged in long-term, outreach-based rehabilitation. Despite formally holding decision-making authority, the occupational therapist enters the organisation as an external actor lacking relational legitimacy within the inner setting (see the Guarding the boundary between insiders and outsiders section). Our interpretation is that this resulted in clashing perspectives, collaborative strain, and, in some cases, even direct conflict. As staff feel a responsibility for managing scarce resources, they must constantly mediate between residents' needs and organisational constraints. Activities are therefore evaluated not only on clinical merit but also on their resource implications. To preserve their identities as competent care providers while resisting additional demands, staff must frame the organisation as constrained. This transforms the implementation of the intervention into a negotiation of competing demands.

One way to understand staff's resistance towards the occupational therapist is therefore not as opposition to improved care but as a discursive strategy for managing competing demands in the organisation. In ELR, the introduction of an occupational therapist is one of the most significant practical changes for staff. As a result, they are often perceived as representatives of the intervention and therefore become suitable targets for criticism. As occupational therapists often advocate for change, their actions may be perceived as a critique of prior practices. Our pre-understanding is that municipalities generally lack formal structures for intersectoral collaboration in supported accommodation. While the distribution of pharmaceutical often guided by clear guidelines, rehabilitation tends to receive less organisational attention. The tension between occupational therapists and staff may therefore be understood as an expression of a broader institutional discourse that privileges biomedical forms of care over recovery-oriented practices.

### 3.5 Blurred accountability, low expectations, and inevitable constraints

The manager was not involved in the everyday work of the staff. Even when staff worried about resources, they were reluctant to request support from the manager. In general, staff seemed unsure about who was responsible for what between themselves, occupational therapists, and managers. These systematic issues result in low expectations and are responded to by framing these problems as inevitable, a positioning that further entrench the status quo.

Excerpt: Interview 0104

Speaker 1: And I also want to underscore this, like the language in the information, that you can choose whatever you want. Here I get, and now I have to speak for myself, it is a little bit frightening for us, for me, when you read something like that. Because then you wonder, will I be able to live up to whatever this individual can come up with. I have to be honest, when it says something like that, that pretty much everything is allowed. Then you get like, “Oh boy, I hope you're not asking for something I'll have to say no to”.

Interviewer: Okey, right. I where thinking… there have been a few management changes for you. And I know that it varies between units, how self-sufficient the units are and so on. But did you feel that you needed more support from management, or did you feel that you got enough support to… manage?

Speaker 2: Yes, if you mean the support, that we would… with staff and so. We talked earlier about economy and that kind of stuff. It wasn't anything there. And well, we are the same staff, we did everything as usual. This was just one additional thing. So, I don't know. What could she (the manager) have done? Should extra people have been brought in?

Speaker 1: I guess it is also a matter of personality. I mean, given the circumstances. Like, she (the manager) had a lot to think about then. If she had remembered this as well, and reminded us about this, “have you noticed that you had a deadline for this?”. That way, we probably would have been less caught off guard in August. But she didn't think of either.

Speaker 1: A little bit like us. I'm just saying, we dropped the ball, she dropped the ball too. But, but… If she had had a bit more focus on that, it might have been easier for us to focus on it a bit more.

Interviewer: It is always different and many units where the manager, or satellite units, where the manager is not on-site, are often very independent.

Speaker 1: She was struggling with some housing issue or something like that, really intensely, sometime before she (the manager) left. And we were, well… yeah, I guess we were. That's right, we were pretty self-sufficient. We were keeping things going quite well.

#### 3.5.1 Text and discursive practice

This excerpt illustrates how little support staff expect from their manager: “So, I don't know. What could she have done? Should extra people have been brought in?”. This rhetorical question signals resignation regarding the potential influence of the manager. By using an interrogative modality (“What could she have done”), the respondent avoids attributing blame and constructs the manager’s potential influence as limited. Logically, the argument is based on the presupposition that additional resources (“extra people”) are unrealistic, reproducing an organisational discourse of resource scarcity and constraints. The intervention is effectively being constructed as a potential threat to organisational stability. The characterisation of the intervention's boundaries as ambiguous (“that pretty much everything is allowed”) is used to justify staffs' anxiety about resource strain and being forced into uncomfortable decisions. Being gatekeepers of collective resources is associated with additional emotional burden (“I hope you're not asking for something I'll have to say no to”). By attributing implementational challenges to the manager's forgetfulness (“she had a lot to think about then”), the issue is reframed as a matter of individual rather than systemic factors. The metaphor “We dropped the ball, and she dropped the ball” is a way for staff to diffuse responsibility and frame implementational issues as a shared failure. Our interpretations are that the manager's absence and the unit's ability to “keep things going” reflect a social practice of resilience, where staff must rely on informal leadership to sustain operational stability.

Staff discourse reveals that the manager often did not actively participate in the implementation process. When asked about support from upper management, one group laughingly said: “Never heard anything about that. I don't think that they even know that we participated (0103).” By expressing their amusement at the question, staff clearly signal low expectations for leadership involvement. Instead, staff placed responsibility for the intervention on the occupational therapist: “I think that is the general critique from us, that the one responsible [the occupational therapist] is not on-site (0301).” This reveals a discursive shift in which accountability for implementation is placed on the occupational therapist, despite the manager's formal responsibility for ensuring adherence. This is not just a pragmatic adaptation but a discursive act that redefines the boundaries of institutional obligations. This is problematic, as the activities delegated by the occupational therapist were often perceived as being assigned without regard for their existing workload: “it became like, here is something you should do, but there was not always time for it (0105).”

Another example of how the occupational therapist became entangled in managerial issues emerged later in the excerpt interview, when the group told a story of a conflict that arose after one of them refused to pay out of pocket during a coffee shop visit with a resident: “Someone probably told the resident, and she mentioned it to the occupational therapist. And then we got into an argument about it. Because suddenly, apparently, we lacked commitment, just because we didn't want to pay out of our own pocket. We also asked her [occupational therapist] if she would have done it (0104).” According to the staff, this refusal was framed by the occupational therapist as a sign of a lack of commitment, an act which the staff perceived as antagonistic. They emphasise that the occupational therapist would never submit to this kind of demand, reflecting a perceived injustice regarding professional status.

When staff was asked how the manager dealt with the situation, the response was somewhat confused: “She didn't say that much at al. It is what it is, we are not getting any extra resources. I think that she [manager] had a lot on her plate right then. Of course she was engaged in us, but she didn't have time to do so much. Like concretely. She was also sick a lot… That was a good question (0104).” Here, the manager is constructed as engaged but lacking the capacity for actual do anything about the problem. Fatalistic language (“it is what it is”) shuts down any critique and frames resource scarcity as inevitable. The use of collective pronouns (“us” and “we”) maintains in-group solidarity and discourages questioning the efforts of the manager. The comment “That was a good question” suggests that these issues are not typically explored. Our interpretation is that the absence and powerlessness of the manager are normalised throughout the corpus, with a lack of management involvement often framed as an unavoidable consequence of circumstance.

#### 3.5.2 Social practice

Our interpretation is that vague leadership and the historical absence of evidence-based methods have fostered a culture of independent practice among staff, in which limited expectations of managerial support have become normalised. Although managers hold formal authority, staff do not rely on them for guidance on how they should approach their work. This would foster a decentralised power dynamic, allowing local opinion leaders to exert strong informal influence on the attitudes of others. It is not unlikely that staff would resist greater managerial involvement, having grown accustomed to and preferring their current autonomy. However, the absence of strong, present leadership creates a vacuum that staff and occupational therapists must fill to maintain organisational stability.

In many participating municipalities, the manager's role in implementing the intervention has been poorly defined. Even though managers were expected to organise regular meetings as part of the intervention, adherence to them has been consistently poor. In practice, responsibility for implementing the intervention has instead fallen entirely to the occupational therapist. This transfer of responsibility has blurred accountability structures, leaving it unclear who bears responsibility for both the implementation process and outcomes. There is a potential risk that having such a strong informal power structure, without an incentive to take risks, fosters an organisational defensiveness about established practices.

## 4 Discussion

In this study, we have explored how discursive and social practices shape cultural norms and how power dynamics influence the uptake of ELR in a real-world setting. Staff working in Swedish-supported accommodation face many contextual challenges, such as limited education, inadequate managerial support, uncertain staffing, transportation difficulties, low status, and stigma. However, listing these contextual factors does not fully explain how they influence the implementation outcomes of recovery-oriented interventions. We identified five discursive representations that show how language, roles, and informal power dynamics influence the enactment of these challenges in practice.

Previous studies have also observed this tendency to equate novel interventions with established practices, with some suggesting that education may even reinforce the belief that recovery is not substantially different from current approaches (65). While this conflation may facilitate organisational buy-in, it diminishes the intervention's capacity to challenge existing norms. Since staff’s understanding of recovery-oriented practice shapes how the intervention is implemented (66), this could affect the effectiveness of the intervention. All participating staff expressed support for the theoretical rationale of ELR (recovery through activity), even though some found the language too difficult. One possible explanation for this conceptual ambguity is that some components were never used, leading to insufficient engagement with the educational material. Although the intervention appears to legitimise some recovery-oriented values within the organisation, many respondents had not fully internalised the material. Greater effort is likely needed to ensure that staff can recognise its relative advantage.

Mental health staff are often ambivalent towards recommendations from national guidelines, fearing that these will conflict with the therapeutic relationship (67). This resembles the emphasis on relational legitimacy we found in our corpus, which is founded on a strong loyalty to the inner setting and a strive for cohesiveness. Nevertheless, despite collaborative issues, all units expressed a desire for increased support from external actors to engage residents in activities. Even though the occupational therapist often filled this role, they were often criticised for lacking relational legitimacy, a dynamic that made the introduction of new perspectives difficult.

Mental health practice is not characterised by ideological consensus, and staff often advocate different models of practice based on their educational and professional experiences (68). Limited understanding of different roles is a common barrier when implementing recovery-oriented interventions (23). There is a risk that the relationship between staff and occupational therapists was perceived as hierarchical rather than collegial, leading to conflicting priorities and making the implementation of recovery-oriented practices difficult (69). To better promote collaboration and effective interprofessional communication, it may be necessary to foster increased understanding and appreciation of each other's professional roles (68).

Increased resident autonomy was consistently framed as a threat to organisational stability. The intervention reallocates decision-making power away from staff, and even if perceived as a minor disruption, it requires the organisation to re-emphasise residents' priorities to be successful (70). Mental health staff currently have substantial control over which decisions residents can influence and when (8). Although relinquishing power can be understood as part of a broader empowerment process, staff experience it as threatening. Since complex systems naturally gravitate toward self-organisation (71), new practices must be continually negotiated in relation to existing needs and constraints.

To manage expectations and prevent residents from pursuing unsuitable goals, interviewed staff adopted a paternalistic discourse. Even when guided by good intentions, this risks reproducing the invisible power structures often found in mental healthcare (72). Struggling to balance the promise of self-determination with its potential impact on collective resources, staff seem to have adopted a defensive and risk-averse approach. Risk management has been identified as one of the leading discourses in mental healthcare, even if it is countertherapeutic in many cases (73). Rather than avoiding risk, recovery-oriented practice often relies on creative risk-taking, where staff must feel encouraged to support residents in exercising their right to take necessary risks (70).

When managers fail to take adequate accountability, the responsibility for distributing resources falls to frontline staff, often leading to defensive behaviours within organisations (74). A reduced focus on managing scarce resources would allow staff to prioritise delivering high-quality services, while addressing economic barriers, such as reimbursement for work-related expenses, could support adoption. Mental health staff tend to expect change from higher organisational levels (67), and the absence of formalised leadership seems to have created space for conflict rather than collaboration between staff and occupational therapists. Interdisciplinary collaboration requires clear role boundaries and lines of accountability between professions to be successful (75, 76).

Even if there is broad agreement on what needs to be done, ambiguity often exists about who is responsible, when it should be done, and how. In our corpus, the responsibility for implementing the intervention had been shifted almost exclusively to the occupational therapist, extending well beyond their scope of practice. To reduce the risk of rivalry, competition, and professional stereotyping, managers need to adopt a much more “hands-on-approach” (75). The organisation also needs to introduce more structured implementation strategies to to sustain the intervention over time. Personal recovery is not about applying a specific model; it is about enacting values. Mental health organisations often function as distinct “cultures of care,” with local contexts exerting a strong influence on how care is understood and delivered (77). Our findings suggest that it is likely necessary to cultivate a stronger sense of ownership among staff to enable them to take greater responsibility for shaping and developing their own practices. Broader cultural change might be needed to ensure an organisation that prioritises quality, embraces creative risk-taking, and complies with legal standards.

### 4.1 Methodological considerations

While our analysis discusses how discourses may have influenced implementation processes and outcomes, the study design does not allow us to establish causal relationships empirically. Rather, the analysis generates theoretically informed hypotheses about how discourse may have influenced the implementation process in this context. Future research is needed to assess these hypotheses through methodological triangulation and piecemeal experimentation, including the use of quantitative measures of implementation outcomes. It is worth noting that while the present study focused only on housing staff discourses, the experiences of occupational therapists, managers, and residents are explored in other studies. Some contextual and linguistic nuances might have been lost while translating excerpts from Swedish to English. All translations were done by the research team, whose members are all non-native English speakers. However, all analyses were conducted in Swedish, limiting the potential impact of translation on the findings.

In the corpus, we observed clear tendencies towards consensus within each housing unit, which may have constrained the expression of certain discourses while reinforcing others. Internal organisational norms and social pressures may have discouraged the expression of dissenting views, resulting in a clustering of perspectives within the units. Our municipal contact persons also had considerable influence over which units were approached for participation in the focus groups, which may have affected the sampling process. In addition, there is a potential risk that staff discourses may have been influenced by the interviewer's affiliation with the research project. To mitigate this risk, the interviewer, who had previous experience as a housing manager, intentionally adopted a collegial approach throughout the focus groups. We believe that this approach helped reduce the extent to which participants associated the interviewer with the research project.

Applying CDA to empirical data, such as interviews, presents a methodological challenge. The primary study object in CDA is naturally occurring text, meaning text produced independently of the research process (78). Since the interview interaction itself is not the object of our analysis, we are not concerned with discourses that emerge solely as a response to the interview setting. Distinguishing discourses that reflect real-world language when analysing transcribed material is challenging. As a result, the production of “text” in the focus groups is less emphasised in our findings. Furthermore, the material consists of retrospective accounts of past experiences, meaning that the discourses identified in the focus groups may not fully reflect those present during the intervention itself. It is also likely that some discourses were not fully captured in the focus groups, as identifying these requires more naturalistic forms of communication.

In addition, the identification and interpretation of discourses are inevitably shaped and influenced by our theoretical assumptions and analytical choices. Alternative interpretations of the corpus are possible, and findings should be understood as one plausible interpretation, rather than a definitive account of the discourses examined. Rather than reporting details in the coding, we sought to ensure transparency by presenting the analytical steps underlying our interpretations through analyses of text, discursive practice, and social practice. Our pre-understanding of the context and intervention may both enrich and influence the findings. The research team brings together experience from social work, implementation and intervention science, psychology, occupational therapy, and public health, and throughout the process, analytical sessions were held to reflect on different interpretations.

It is important to acknowledge that ML is the developer of ELR and the principal investigator of the whole research project. However, she was not involved in data collection, which was conducted by AS, who also led the data analysis. As this study is conducted after a successful effect evaluation (79), the corpus was interpreted from the supposition that ELR is, and has been, an effective and beneficial intervention. The interviewers were blinded to the outcomes of each unit, and all transcripts were analysed using the same analytical approach regardless of trial outcomes. This reduces the risk of confirmation bias and selectively highlighting discourses from particular units, contexts, or outcomes. Consequently, units were not systematically grouped in the analysis. While this strengthens the independence of the analytical process, it may have limited our ability to identify discourses associated with specific implementation processes or outcomes.

## 5 Conclusion

The analysis shows that exploring discourses can be useful for understanding how implementational barriers emerge and are sustained in supported accommodation. Based on the corpus, our interpretation is that staff operate within an organisational culture characterised by cohesiveness, in which relational legitimacy appears to be the primary criterion for inclusion in the inner setting. This type of culture inherently creates tensions when attempting to integrate external actors, such as occupational therapists, into the organisation. Another interpretation is that blurred accountability structures concerning implementation and resource management further intensified this tension, ultimately leading staff to frame residents' self-determination as a potential threat to organisational stability.

Despite the success of the intervention and staff-expressed need for additional external support, the organisation seems to remain passive, effectively relinquishing ownership of the implementation process to the occupational therapist. Often, staff did not seem to resist the intervention itself but rather the way was framed and implemented within the organisation. Addressing this issue requires managers to take deliberate steps to bridge organisational and local discourses by clarifying shared priorities and reframing resident autonomy as an organisational priority. In addition, formal structures should be developed to establish occupational therapists as legitimate collaborative partners, enabling them to work alongside staff as insiders. We argue that without greater managerial involvement, implementation responsibility will continue to be delegated, leaving the discursive tensions that hinder successful implementation largely unresolved.

## Data Availability

The datasets presented in this article are not readily available because the dataset supporting its conclusions includes sensitive personal data collected from a restricted area and thus is not publicly available. The data that support the findings of this study may be made available by the principal investigator, ML, upon reasonable request. Requests to access the datasets should be directed to maria.lindstrom01@umu.se.
